# A comparative study on the effects of boiling and ultrasonication on radical scavenging activity, casein particle size, and whiteness of milk

**DOI:** 10.14202/vetworld.2021.1784-1787

**Published:** 2021-07-09

**Authors:** Tanmay Hazra, Rohit Sindhav, Ch. V. K. Sudheendra, Mitul Bumbadiya, Radhika Govani, Vimal Ramani

**Affiliations:** 1 Department of Dairy Chemistry, College of Dairy Science, Kamdhenu University, Amreli, Gujarat, India; 2 Department of Dairy Technology, College of Dairy Science, Kamdhenu University, Amreli, Gujarat, India; 3 Department of Dairy Microbiology, College of Dairy Science, Kamdhenu University, Amreli, Gujarat, India; 4 Department of Dairy Business Management, College of Dairy Science, Kamdhenu University, Amreli, Gujarat, India

**Keywords:** antioxidant, radical scavenging activity, skim milk, ultrasonication

## Abstract

**Background and Aim:**

Different processing treatments affect the functional properties of milk. This study aimed to evaluate the effects of boiling and ultrasonication on radical scavenging activity, micellar casein particle size, and the whiteness of milk.

**Materials and Methods:**

Milk was subjected to boiling and ultrasonication treatments. Then, the micellar casein size and the whiteness of the milk were evaluated using L-Value- intensity of whiteness and the radical scavenging capacity of the milk was evaluated using 1,1-diphenyl-2-picrylhydrazyl method.

**Results:**

The radical scavenging activity of the milk was found to be reduced during the different processing treatments, but this decrease was non-significant for the ultrasonication treatment (p>0.05). However, a significant reduction in radical scavenging activity (p<0.05) was observed for the boiled milk. Micellar casein size reduction was observed after both treatments, and boiling had a significant effect (p<0.05) on the micellar casein particle size. We found that the whiteness of skim milk was significantly decreased after boiling treatment, but was not significantly decreased after ultrasonication. Ultrasonication had a non-significant effect on the whiteness of ultrasonicated milk which was observed.

**Conclusion:**

Ultrasonicated milk had a very non-significant effect on the antioxidant activity (radical scavenging activity) of milk, whereas the effect of boiling was significant. Ultrasonication treatment increases the shelf-life of milk while retaining its bioactive properties.

## Introduction

Milk is not only a stable source of high-quality protein, lipids, and carbohydrates but is also a prominent source of various bioactive substances that are essential for human growth and immunity [1,2]. Worldwide, cow, buffalo, goat, and sheep milk are widely consumed and used for culinary purposes. Water is the prominent substance in milk, making up 80-85% of the total volume. Due to its high water quantity and the presence of high-quality nutrients, milk is an ideal medium for complex microbial ecosystems; however, these microbes adversely affect the quality of milk during storage and processing [3,4]. Due to the highly perishable nature of milk, maintaining milk without changes to its nutritional, safety, or functional properties is a challenging task. Heat treatment (e.g. boiling, pasteurization, and thermization) is widely used to enhance the keeping quality of milk [4,5]; however, heat treatments directly affect the functional and nutritional qualities of milk, especially milk proteins [6].

Milk proteins (casein and whey) play essential roles in the processing and functional properties of a variety of dairy products. The antioxidant and radical scavenging activity of milk has been well documented [7], and milk proteins play a significant role in the antioxidant activity of milk [7,8]. In this current era, consumers are aware of the nutritional profiles of foods; therefore, adverse changes in milk due to heat processing are undesirable. To address these concerns, milk processors are now seeking novel, non-thermal processing methods, such as ultrasonication treatment.

During ultrasonication treatment, acoustic cavitation is produced, and this treatment has been successfully applied to process milk and dairy products [9]. The effect of ultrasonication on the reduction of bacterial load has been demonstrated in the previous study [10]. However, Lin *et al*. [11] observed morphological changes in casein during ultrasonication.

The effect of ultrasonication on milk antioxidant activities has not been fully investigated; therefore, in this study, we aimed to ascertain the effects of boiling and ultrasonication on the physicochemical and antioxidant properties of milk.

## Materials and Methods

### Ethical approval

Ethical approval was not necessary for this study as milk was procured from farmers.

### Study period and location

This study was conducted in the month of September 2020 at College of Dairy Science, Amreli, Gujarat

### Procurement of milk

Cow milk was procured from local farmers in the Amreli district and was skimmed by centrifugation at 3500 rpm for 45 min at 4°C.

### Compositional analysis

The proximate composition of the cow skim milk (CSM) samples was analyzed using the MilkoScan™ Mars instrument (Foss analytics- Hilleroed, Denmark).

### Processing of milk

CSM samples were boiled at 100°C for 15 min. For ultrasonication treatment, milk samples were sonicated at 20 kHz at 150 W for 10 min (JY92-IIN). The samples were equilibrated at room temperature (25°C) for 20 min before measuring the parameters.

### Measurement of particle size of CSM

Casein micelle sizes were determined using a Zetasizer- Malvern Pan analytical- Delhi, India. The viscosity and refraction index of water were 0.8872 cP and 1.330, respectively. For each sample, the light scattering measurements were carried out at 25°C.

### Measurement of milk whiteness

The degree of change in whiteness was measured using a Hunter colorimeter (Color Flex EZ colorimeter) and represented as an L* value (representing lightness). The instrument was standardized with a standard reference (scale 1-100). Higher score identified higher whiteness intensity.

### 1,1-Diphenyl-2-picrylhydrazyl (DPPH) radical scavenging activity of milk

The free radical scavenging activities of the milk samples were measured using the DPPH method described by Mann *et al*. [12]. This assay is generally performed to determine the antioxidant activity in biological systems.

### Statistical analysis

Data were presented as the mean±standard error of the mean. Analysis of variance was used to perform statistical comparisons between all the treatments, where p<0.05 was considered statistically significant. Graphs were prepared using Microsoft Excel version 2010.

## Results

### Overall composition

The overall composition of the milk is shown in Table-1. Solid not fat made up 8.78±0.21% of the milk, lactose accounted for 4.28±0.06% of the overall composition, and protein made up 4.1±0.07% of the overall composition.

**Table-1:** The overall composition of cow skim milk.

Constituents	Value
SNF	8.78±0.21
Lactose	4.28±0.06
Protein	4.1±0.07

SNF=Solid not fat

### Effect of processing on micellar casein size

**Figure-1:**
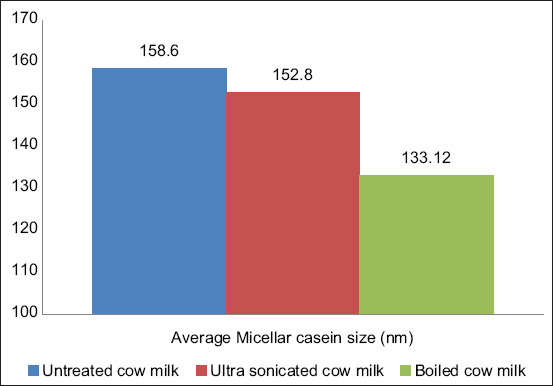
The average size of micellar casein (nm) of cow milk.

The effect of processing on the particle size of micellar casein in the milk is presented in Figure-1. The average size of micellar casein in the raw cow milk was found to be 158.6±2.20 nm, whereas the average size was 152.8±1.34 nm in the ultrasonicated milk and 133.12±0.14 nm in the boiled milk, a significant reduction (p<0.05) was observed for boiled milk only.

### Effect of processing on the whiteness of CSM

**Figure-2:**
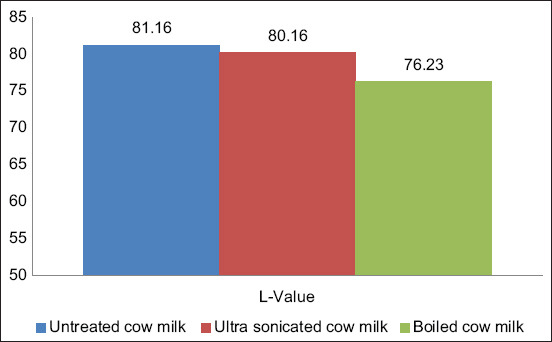
The average L-value (whiteness) of cow milk.

The degree of change in whiteness was measured using a Hunter colorimeter, and the results are presented in Figure-2. The average whiteness (L value) of raw cow milk was found to be 81.16±0.56. The effect of ultrasonication on the whiteness of the milk was found to be non-significant (p>0.05), with an L value of 80.16±0.12 nm. However, boiling treatment significantly (p<0.05) reduced the milk whiteness (76.23±0.10) by almost 7%.

### Effects of processing on radical (DPPH) scavenging activity of milk

**Figure-3:**
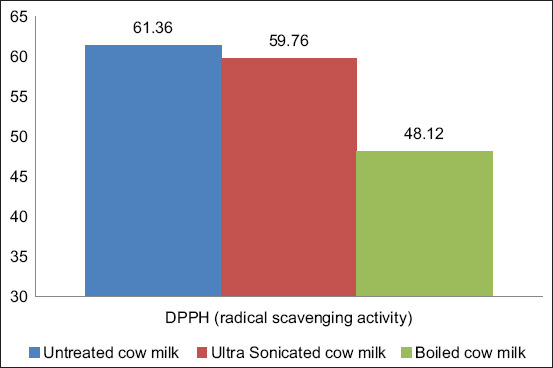
The average DPPH (radical scavenging activity) of cow milk.

The effects of processing on radical (DPPH) scavenging activity in milk are presented in Figure-3. The radical scavenging activity of cow milk, boiled cow milk, and ultrasonicated cow milk was 61.36±1.45%, 48.12±2.36%, and 59.75±0.86%, respectively. It was observed that, for both processing treatments, the antioxidant activities of milk in terms of radical (DPPH) scavenging activity were reduced, but in the case of boiling, the reduction was significant (21.50%, p<0.05), whereas the reduction was not significant (2.60%, p>0.05) in the case of ultrasonication.

## Discussion

Milk is a complex biochemical fluid and an ideal medium for the growth of various microorganisms. Therefore, maintaining the quality of milk is a challenging task. In addition to the traditional heat treatments, dairy producers are now considering alternative non-thermal processing methods, such as ultrasonication treatment, to enhance shelf-life while retaining the nutritional and biofunctional properties of milk [5].

Casein plays an essential role in terms of both processing and the functional properties of milk [2,7]. Heat treatment modifies the structure of micellar casein: Dephosphorylation and precipitation of calcium can be observed during treatment at high temperatures. In this study, it was observed that micellar casein particles were reduced in size during heating, in line with the earlier observation of Yang *et al*. [13] who also reported that the average size of micellar caseins was reduced after heat treatment. Liu *et al*. [14] also reported that, during heating, shrinkage of micellar casein occurred and the sizes of casein particles were reduced. Ultrasonication creates mechanical wavelengths that disrupt micellar casein; therefore, micellar casein size is usually reduced during this process [11]. Indeed, the results of the current study showed that micellar casein particle size was reduced during ultrasonication treatment. Yang *et al*. [15] reported that the secondary structure of proteins in skim milk changes during ultrasonication treatment and that the size of casein particles is reduced. The size reduction of micellar casein due to ultrasonication might be due to disruption of casein or changes in its structure. However, our findings suggest that the effect of heat on the disruption of micellar casein particles was more intense than that of ultrasonication treatment.

The color of milk is usually white, and this whiteness is due to the reflection of light by fat globules and micellar casein. Beta-carotene also affects the color of milk [16] and creates a yellowish hue. We observed a significant reduction in the whiteness of milk during boiling treatment. Degradation and structural modification of casein occurs during heat treatment and may account for the observed reduction in L value for the boiled milk in this study. We also observed a non-significant effect on the whiteness of the milk after ultrasonication treatment. However, the previous studies have reported no observed effect on milk color during ultrasonication treatment [17].

The DPPH assay is used to determine antioxidant or free radical scavenging activity in biosystems [12]. Milk is an ample source of antioxidant compounds that often change during mechanical or thermal treatment [1]. In our study, it was observed that both heat treatment and ultrasonication treatment reduced the radical scavenging activities of milk. Several previous studies have reported that heat treatment reduced the radical scavenging activities of milk [18]. Casein has an effective role against lipid peroxidation and in radical scavenging activity [7,8,19]. Micellar casein is denatured during heating; therefore, the radical scavenging activity of heat-treated milk is reduced compared to non-treated milk. In the case of ultrasonication treatment, the denaturation of micellar casein was not as severe; therefore, the radical scavenging activity was higher compared to heat-treated milk.

## Conclusion

The antioxidant activity in milk changes during boiling and ultrasonication treatment, and boiling treatment confers a greater reduction in antioxidant activity compared to ultrasonication. Higher micellar casein degradation was observed in boiled milk than in ultrasonicated milk. In view of the esthetic quality of milk, higher whiteness was observed in ultrasonicated milk samples as compared to boiled milk samples. Our results reveal that the application of ultrasonication treatment provides superior results over boiling treatment in terms of the esthetic and bioactive qualities of milk, as well as increasing the shelf-life.

## Authors’ Contributions

TH: Conceptualized, performed the study, and drafted the manuscript. RS and MB: Execution of the project and drafted the manuscript. VR: Planning of study and drafted the manuscript. ChVKS: Edited the manuscript. RG: Data analysis and edited the manuscript. All authors read and approved the final manuscript.
